# The use of modified TI-RADS using contrast-enhanced ultrasound features for classification purposes in the differential diagnosis of benign and malignant thyroid nodules: A prospective and multi-center study

**DOI:** 10.3389/fendo.2023.1080908

**Published:** 2023-02-01

**Authors:** Ping Zhou, Feng Chen, Peng Zhou, Lifeng Xu, Lei Wang, Zhiyuan Wang, Yi Yu, Xueling Liu, Bin Wang, Wei Yan, Heng Zhou, Yichao Tao, Wengang Liu

**Affiliations:** ^1^ Department of Ultrasound, The Third Xiangya Hospital, Central South University, Changsha, Hunan, China; ^2^ Department of Ultrasound, Yiyang Central Hospital of Hunan University of Chinese Medicine, Yiyang, Hunan, China; ^3^ Department of Ultrasound, Shenzhen Second People’s Hospital, Shenzhen, Guangdong, China; ^4^ Department of Ultrasound, Huang Shi Central Hospital, Huang Shi, Hubei, China; ^5^ Department of Ultrasound, Hunan Cancer Hospital, Changsha, Hunan, China; ^6^ Department of Ultrasound, The People’s Hospital of Liuyang, Changsha, Hunan, China; ^7^ Department of Ultrasound, The First Affiliated of Guangxi University of Chinese Medicine, Nanning, Guangxi, China; ^8^ Department of Ultrasound, Yueyang Central Hospital, Yueyang, Hunan, China; ^9^ Department of Ultrasound, Hubei Provincial Hospital of Traditional Chinese Medicine, Wuhan, Hubei, China; ^10^ Department of Ultrasound, Xiaogan Central Hospital, Xiaogan, Hubei, China

**Keywords:** thyroid, thyroid imaging report and data system, contrast-enhanced ultrasound, prospective, multi-center

## Abstract

**Objectives:**

To evaluate the diagnostic efficacy of a modified thyroid imaging reporting and data system (TI-RADS) in combination with contrast-enhanced ultrasound (CEUS) for differentiating between benign and malignant thyroid nodules and to assess inter-observer concordance between different observers.

**Methods:**

This study included 3353 patients who underwent thyroid ultrasound (US) and CEUS in ten multi-centers between September 2018 and March 2020. Based on a modified TI-RADS classification using the CEUS enhancement pattern of thyroid lesions, ten radiologists analyzed all US and CEUS examinations independently and assigned a TI-RADS category to each thyroid nodule. Pathology was the reference standard for determining the diagnostic performance (accuracy (ACC), sensitivity (SEN), specificity (SPN), positive predictive value (PPV), and negative predictive value (NPV)) of the modified TI-RADS for predicting malignant thyroid nodules. The risk of malignancy was stratified for each TI-RADS category-based on the total number of benign and malignant lesions in that category. ROC curve was used to determine the cut-off value and the area under the curve (AUC). Cohen’s Kappa statistic was applied to assess the inter-observer agreement of each sonological feature and TI-RADS category for thyroid nodules.

**Results:**

The calculated malignancy risk in the modified TI-RADS categories 5, 4b, 4a, 3 and 2 nodules was 95.4%, 86.0%, 12.0%, 4.1% and 0%, respectively. The malignancy risk for the five categories was in agreement with the suggested malignancy risk. The ROC curve showed that the AUC under the ROC curve was 0.936, and the cutoff value of the modified TI-RADS classification was >TI-RADS 4a, whose SEN, ACC, PPV, NPV and SPN were 93.6%, 91.9%, 90.4%, 93.7% and 88.5% respectively. The Kappa value for taller than wide, microcalcification, marked hypoechoic, solid composition, irregular margins and enhancement pattern of CEUS was 0.94, 0.93, 0.75, 0.89, 0.86 and 0.81, respectively. There was also good agreement between the observers with regards to the modified TI-RADS classification, the Kappa value was 0.80.

**Conclusions:**

The actual risk of malignancy according to the modified TI-RADS concurred with the suggested risk of malignancy. Inter-observer agreement for the modified TI-RADS category was good, thus suggesting that this classification was very suitable for clinical application.

## Introduction

With the development and wide application of high resolution ultrasound, the detection rate of thyroid nodules has increased significantly (1–3). Although there is a high prevalence of thyroid nodules, only 1.6% to 12% of these are malignant (4, 5). According to the bethesda classification system, only 3%−7% of the thyroid nodules undergoing fine-needle aspiration (FNA) have clearly malignant features, at least 60%–70% of thyroid nodules are proven to be benign *via* pathological analysis. The pathological type of thyroid nodules directly affects the treatment and prognosis of patients. Therefore, the accurate judgment of benign and malignant thyroid nodules is of important clinical significance.

Ultrasound (US) is a simple and reproducible non-invasive method and remains the modality of choice for patients with thyroid nodules. US can distinguish benign and malignant tumors by specific ultrasound imaging characteristics (6, 7). Usually, the suspicious signs of malignant nodules on US include a solid composition, a taller shape rather than a wider shape, an irregular margin, micro-calcification, and marked hypo-echogenicity (8, 9). However, the grey scale and Doppler US features of benign and malignant nodules overlap. Furthermore, a single ultrasound sign cannot reliably predict benign and malignant thyroid nodules (10). Therefore, prediction models have been developed for malignancy that combine multiple US features to improve the accuracy of diagnosing benign and malignant thyroid nodules. In 2009, Horvath et al. were the first to classify thyroid nodules based on the principles that have been used in the breast imaging reporting and data system of the American College of Radiology using ten malignant-related ultrasound features, and proposed the first thyroid imaging reporting and data system (TI-RADS) classification system (11). As a quantitative system for the risk stratification of malignant tumors in thyroid nodules, however, this sonographic model is not applicable to all thyroid nodules and is difficult to apply. In the same year, Park et al. proposed a multi-factor logistic regression analysis equation to predict the malignant probability of thyroid nodules based on 12 types of sonographic features (12). However, this prediction equation is more complicated and difficult to apply. To overcome these limitations, Kwak et al. used several suspicious sonographic features and calculated the fitting probability of malignant tumors (13). The Park equation and the Horvath TI-RADS includes more suspicious malignant features and sonographic patterns and are relatively complex. The model proposed by Kwak et al. simplified the number of suspicious malignant signs on US to five. Subsequently, the TI-RADS became widely used in clinical practice.

Over recent years, the development of new US technology has improved the diagnostic accuracy of thyroid nodules, especially the application of contrast-enhanced ultrasound (CEUS). CEUS can be applied non-invasively in real-time and continuously evaluate the perfusion of the microvessels in thyroid nodules under high frequency US (14). Studies have shown that the combination of the TI-RADS classification with CEUS significantly improves the diagnostic accuracy of thyroid nodules (15, 16). However, CEUS enhancement features for thyroid nodules has still not been fully implemented with the TI-RADS system. In 2017, we proposed a new classification standard based on the TI-RADS classification criteria proposed by Horvath et al. (11), Park et al. (12) and Kawk et al. (13), that combined the five ultrasound signs proposed by Kawk TI-RADS (13), and combined it with CEUS enhancement to form a modified version of TI-RADS (17).

We showed that the modified version of TI-RADS significantly increased diagnostic accuracy for the identification of thyroid nodules, particularly for TI-RADS 4a and 4b lesions. This modified version of TI-RADS was validated by a single center, but all cases were retrospective studies. Thus, the aim of the current study was to confirm the diagnostic efficacy and assess the inter-observer agreement for thyroid nodule characterization using the modified version of TI-RADS in prospective, multi-center trial.

## Materials and methods

This prospective multi-center study was approved by the Institutional Review Boards of the ten participating centers

### Study population

**Figure 1:**
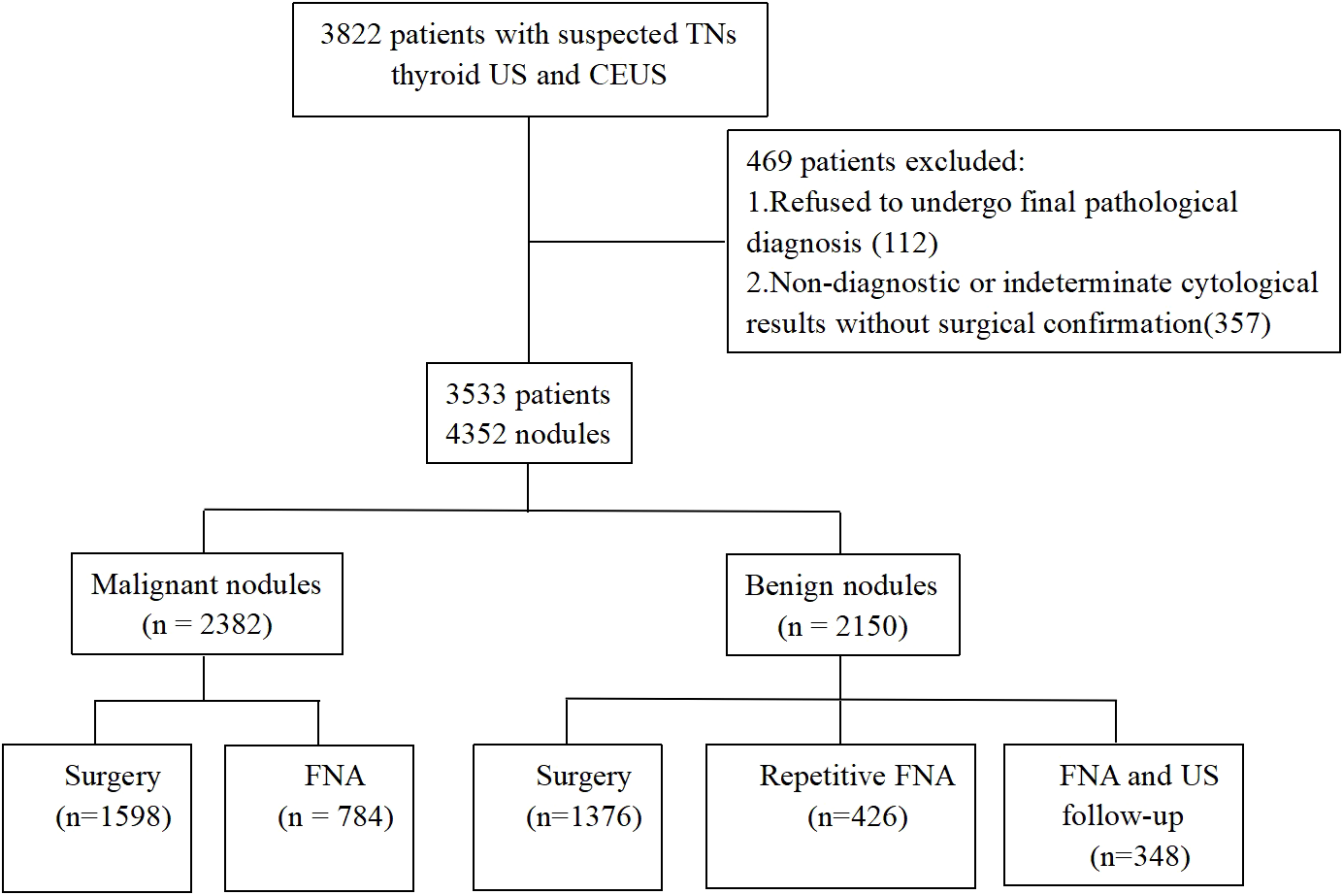
Flow chart of the study population. TNs, thyroid nodules; US, ultrasound; CEUS, contrast-enhanced ultrasound; FNA, fine needle aspiration.

Between September 2018 and March 2020, we initially collected 3822 consecutive patients from ten centers. The inclusion criteria were as follows: (1) patients with clinically suspected thyroid nodules, (2) patients who consented to undergo CEUS and (3) patients with a final pathological diagnosis as determined by surgical pathology or cytopathological results based on the Bethesda system. Patients were excluded if they refused to undergo final pathological diagnosis or had non-diagnostic or indeterminate cytological results for a lesion without surgical confirmation. Finally, our study featured 3353 patients with 4532 thyroidal nodules. Of the 3353 participants, 729 were male and 2624 were female, patient age ranged from 18 to 82 years with a mean of 46.1 ± 12.2 years). The flow chart of our study is illustrated in **Figure 1**.

### Conventional ultrasound and contrast-enhanced ultrasound examination

All patients underwent conventional ultrasound examinations and CEUS analysis. For conventional US examination, we used a linear, high-frequency probe. Patients were positioned in a supine position with dorsal flexion of the head. Thyroid nodules were then evaluated for location, size, echogenicity, internal composition, margin, shape and the presence/absence of micro-calcification. The internal component of each nodule was classified as solid, mixed or cystic. Echogenicity was classified as hyper-echogenicity, iso-echogenicity, hypo-echogenicity or marked hypo-echogenicity. The margins were classified as irregular or regular. Calcifications, when present, were categorized as micro-calcification (equal to or < 1mm in diameter) or macrocalcification (> 1mm). If a nodule showed both microcalcification and macrocalcification, it was classified as microcalcification. Shape was categorized as taller than wide (greater in its anteroposterior dimension than in its transverse dimension) or wider than tall.

Before starting the multi-center study, all hospitals participating in the center were trained on the specific classification methods and standards to establish a unified approach. Ten experienced radiologists used the TI-RADS classification criteria to classify thyroid nodules according to five ultrasound signs (solid component, marked hypo-echogenicity, taller than wide shape, microcalcification and irregular margin) to evaluate each nodule. This was performed in a blind and independent manner. The TI-RADS classification criteria were as follows (17): TI-RADS score 1: normal thyroid; TI-RADS score 2: no malignant sign, benign lesions; TI-RADS score 3: one malignant sign, high probability of being benign; TI-RADS score 4a: two malignant signs, possibly benign; TI-RADS score 4b: three malignant signs, high probability of malignancy; TI-RADS score 5: four to five malignant signs, highly suggestive of malignancy.

### Modified TI-RADS diagnostic criteria in combination with CEUS

The contrast agent used in this study was SonoVue (Bracco, Milan, Italy). A 20-G needle was inserted into the peripheral veins to establish intravenous access. Twenty-five mg of SonoVue was diluted in 5 mL of saline and vibrated for 30s to create a microbubble suspension. The suspension was then injected as a bolus and each 2.4 mL injection was then flushed with 5 mL of saline. The dynamic perfusion of the lesion was continuously observed in real time. The CEUS diagnostic criteria were divided into circular enhancement, high enhancement, equal enhancement and low enhancement when compared to the surrounding thyroidal parenchyma.

**Figure 2:**
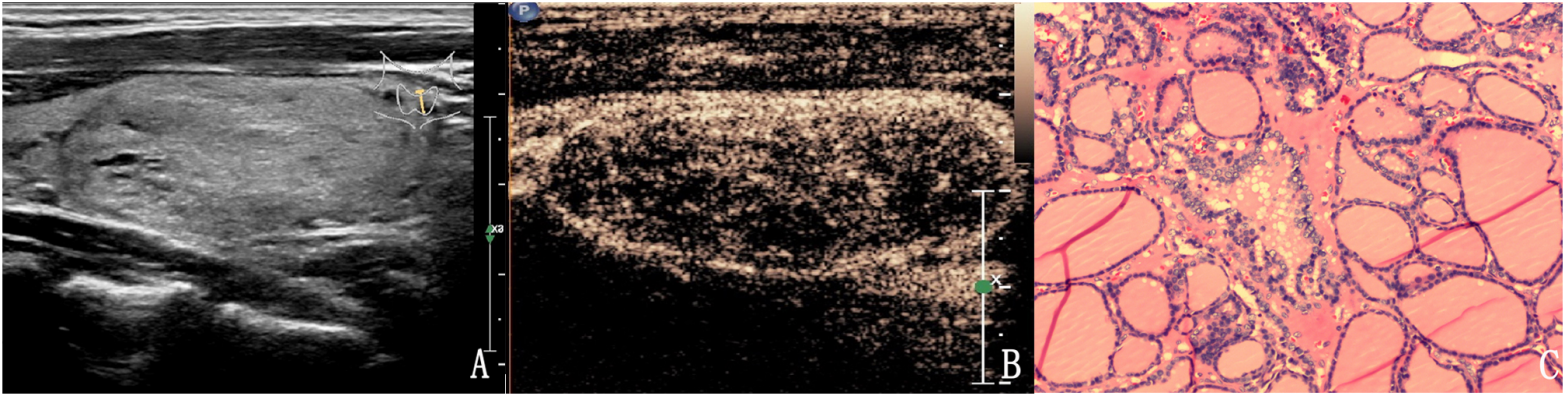
The case of a 46-year-old woman with a 37.3 × 18.1 × 24.7 mm solid hyper-echoic nodule in the left lobe of the thyroid. **(A)** Conventional two-dimensional image showed that the nodule had one malignant indicator (solid) and was classified with a TI-RADS score of 3. **(B)** Ultrasound contrast image showing ring enhancement. The modified version of TI-RADS combined with CEUS returned a score of 2 and the patient was diagnosed with a benign nodule. **(C)** Pathological image of the lesion, a nodular goiter.

**Figure 3:**
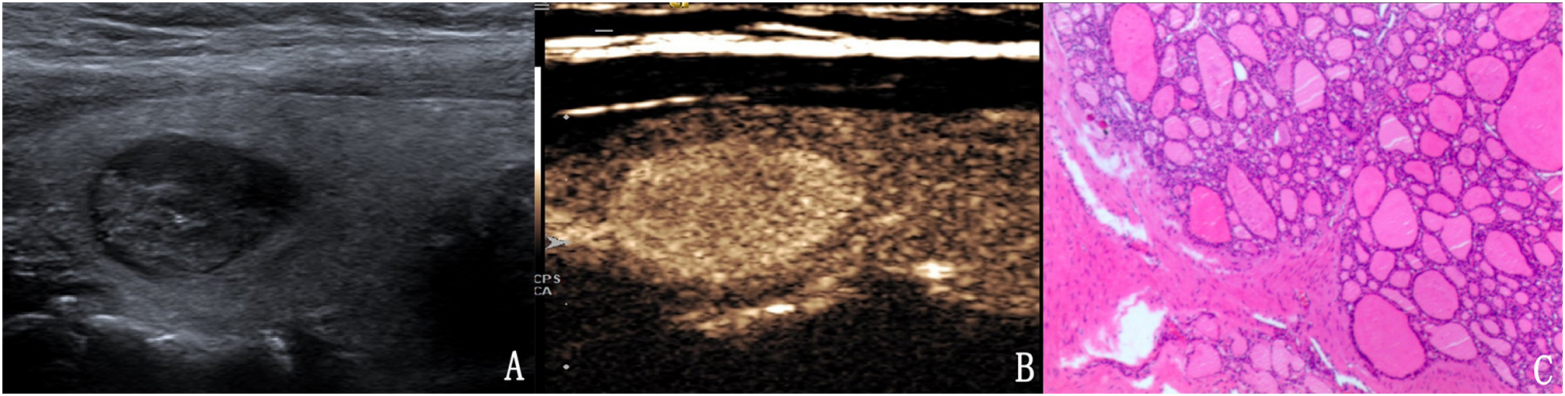
The case of a 31-year-old woman with a 15.1 × 10.1 × 9.8 mm solid hypo-echoic nodule in the right lobe of the thyroid. **(A)** Conventional ultrasound showed that the nodule had one malignant indicator (solid) and was classified as a TI-RADS score of 3. **(B)** Contrast-enhanced ultrasound detected high enhancement. The modified TI-RADS resulted in a score of 2 and indicated a benign nodule. **(C)** Pathology of the lesion showed an adenoma.

**Figure 4:**
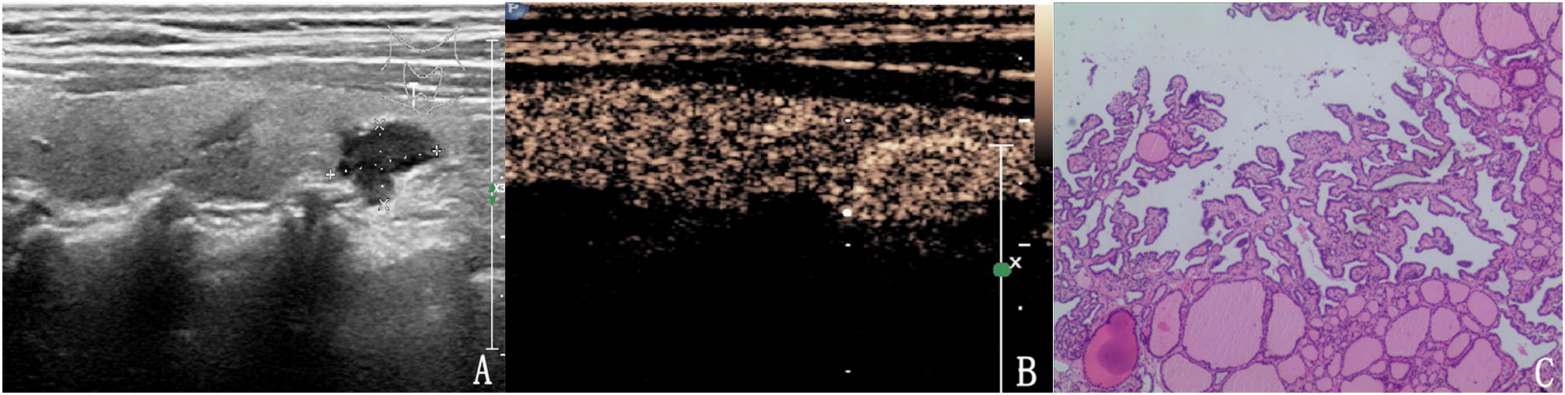
The case of a 64-year-old woman with a 10.6 × 6.7 × 5.9 mm solid mark hypo-echoic nodule in the right lobe of the thyroid. **(A)** Conventional two-dimensional image showing that the nodule had three malignant indicators (solid, mark hypo-echoic and irregular margin) and was classified with a TI-RADS score of 4b. **(B)** Ultrasound contrast image showing high enhancement. The modified version of the TI-RADS combined with CEUS returned a score of 4a, and the diagnosis was a benign nodule. **(C)** Pathological image of the lesion, a nodular goiter.

**Figure 5:**
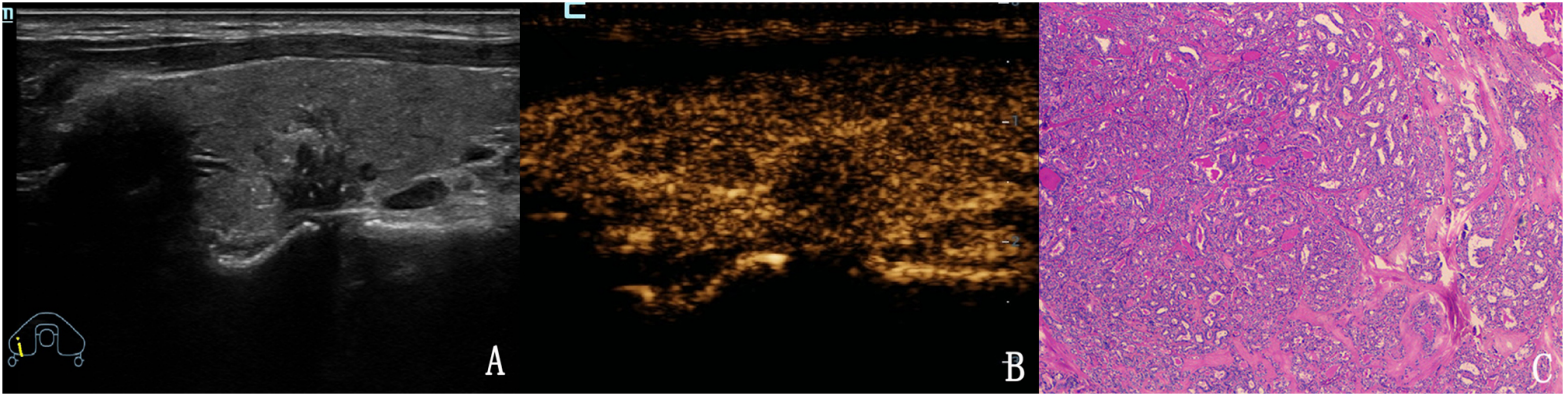
The case of a 43-year-old woman with a 15.2 × 11.1 × 12.5 mm solid hypo-echoic nodule in the right lobe of the thyroid. **(A)** Conventional two-dimensional image showing that the nodule had three malignant indicators (solid, irregular margin and microcalcifications) and was classified with a TI-RADS score of 4b. **(B)** Ultrasound contrast image showing low enhancement. The modified version of TI-RADS combined with CEUS returned a score of 5, and the patient was diagnosed with a malignant nodule. **(C)** Pathological image of the lesion, a PTC.

**Figure 6:**
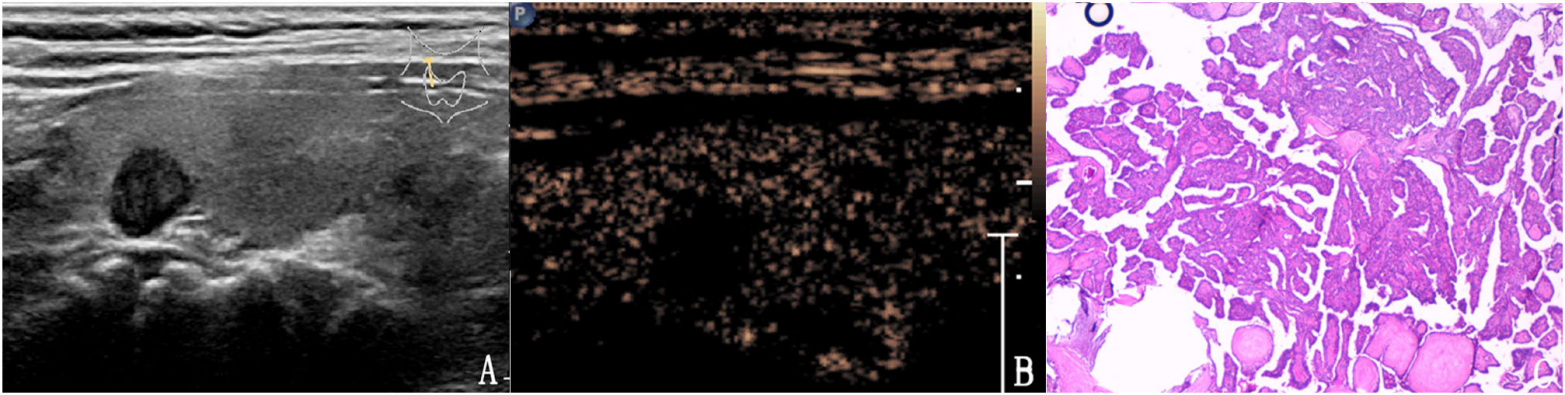
The case of a 47-year-old man with a 7.8 × 6.8 × 7.5 mm solid mark hypo-echoic nodule in the right lobe of the thyroid. **(A)** Conventional two-dimensional image showing that the nodule had two malignant indicators (solid, mark hypoechoic) and was classified with a TI-RADS score of 4a. **(B)** Ultrasound contrast image showing low enhancement. The modified version of the TI-RADS combined with CEUS returned a score of 4b and the diagnosis was a malignant nodule. **(C)** Pathological image of the lesion, a PTC.

**Figure 7:**
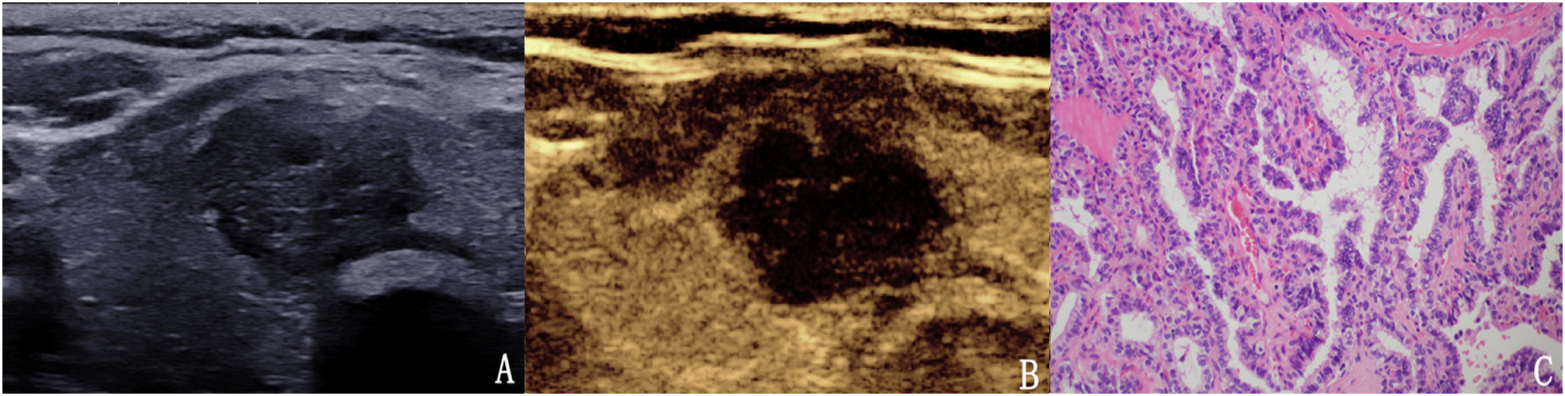
The case of a 51-year-old woman with a 20.4 × 15.1 × 16.9 mm solid mark hypo-echoic nodule in the right lobe of the thyroid. **(A)** Conventional two-dimensional image showing that the nodule had three malignant indicators (solid, mark hypo-echoic and irregular margin) and was classified with a TI-RADS score of 4b. **(B)** Ultrasound contrast image showing low enhancement. The modified version of the TI-RADS combined with CEUS returned a score of 5, and the diagnosis indicated a malignant nodule. **(C)** Pathological image of the lesion, a PTC.

If CEUS indicated high enhancement or circular enhancement, then the TI-RADS score was reduced by one, if the initial score was 2, then the score remained the same (**Figures 2**–**4**). If CEUS indicated low enhancement, then the TI-RADS score was increased by one, a score of 5 remained the same (**Figures 5**–**7**). If CEUS indicated equal enhancement, then the TI-RADS classification remained the same. With regards to the modified TI-RADS classification, scores 2-4a were diagnosed as benign and scores 4b-5 were diagnosed as malignant (**Table 1**).

**Table 1:** Modified TI-RADS diagnostic criteria in combination with CEUS.

Modified TI-RADS classification	Definition	Risk of malignancy	Recommended
TI-RADS 2	benign lesions	0	Long-term follow-up
TI-RADS 3	high probability of benignity	<5%	Short-term follow-up
TI-RADS 4a	possible benignity	5~15%	FNA
TI-RADS 4b	high probability of malignancy	15~90%	FNA
TI-RADS 5	highly suggestive of malignancy	>90%	Clinical treatment

### US-guided FNA procedures

US-guided FNA was performed with 23-gauge needles, each lesion was aspirated three times. Materials obtained from aspiration biopsy were expelled onto glass slides and smeared. All smears were placed immediately in 95% alcohol for Papanicolaou staining. The interpretation of FNA was based on the Bethesda system for reporting thyroid cytopathology.

### Statistical analysis

Statistical analyses were performed using SPSS for Windows software (version 23.0, SPSS, Chicago, III, USA). Measurement data are given as mean ± standard deviation and count data are given as percentage and frequency. Analysis of receiver operating characteristic (ROC) curves were used to determine the cut-off value, area under the curve (AUC), and 95% confidence interval (CI). We calculated the ACC, SEN, SPN, PPV and NPV of the modified TI-RADS system to identify malignant thyroid nodules. The level of significance was defined as p < 0.05. Cohen’s Kappa (κ) coefficient was determined separately to evaluate inter−observer agreement for each of the TI-RADS malignant features. The κ values were interpreted as follows: 0.01–0.20 (poor agreement), 0.21–0.40 (fair agreement), 0.41–0.60 (moderate agreement), 0.61–0.80 (good agreement) and 0.81–1.0 (very good agreement).

## Results

### Nodule diagnosis

The final diagnosis of the 4532 nodules was benign in 2150 (47.4%) nodules and malignant in 2382 (52.6%) nodules. Final diagnoses were determined by surgical resection in 1598 of the 2382 malignant nodules, including 1529 papillary thyroid carcinomas (PTC), 39 follicular carcinomas, 14 cases of focal canceration of nodular goiter, 7 medullary thyroid carcinomas (MTC), 4 anaplastic thyroid carcinomas (ATC), 3 metastatic carcinomas and 2 lymphomas. In total, 784 of the malignant nodules diagnosed by FNA were PTC. The 1376 surgically confirmed benign nodules included 186 adenomas, 990 nodular goiter, 148 Hashimoto’s thyroiditis, and 52 cases of subacute thyroiditis. Overall, 774 benign nodules were diagnosed based on repetitive benign FNA results or benign FNA results by US follow-up studies.

### US features of thyroid nodules

The mean maximum diameter of nodules was 13.6 ± 11.8 mm (range: 4.5–59.0 mm) and the mean size of benign nodules was 18.4 ± 14.0 mm, this was significantly larger than that of malignant nodules (9.3 ± 7.1 mm, p < 0.001). Of the 4532 thyroid nodules, there were 3673 solid composition nodules and 859 mixed composition nodules, 413 marked hypoechoic nodules, 2021 hypoechoic nodules, 473 isoechoic nodules and 1625 hyperechoic nodules. We identified 1453 nodules with irregular margins and 3079 nodules with regular margins, 1733 nodules with microcalcifications, 341 nodules with macrocalcifications and 2458 nodules with no calcifications. There were 3339 wider than tall nodules and 1193 taller than wide nodules (**Table 2**).

**Table 2:** US features of thyroid nodules.

US features	Total lesions	Benign lesions	Malignant lesions	*P value*
Composition				<0.001
Solid	3673	1357	2316	
Mixed	859	813	46	
Echogenicity				<0.001
Marked hypoechoic	413	152	261	
Hyper/iso/hypoechoic	4119	2018	2101	
Margin				<0.001
Irregular	1453	217	1236	
Regular	3079	1953	1126	
Calcification				<0.001
Microcalcifications	1733	439	1294	
Macrocalcifications/no calcifications	2799	1731	1068	
Shape				<0.001
Taller than wide	1193	210	983	
Wider than tall	3339	1960	1379	

### Malignancy risk according to category in the modified TI-RADS

Of the 4352 thyroid nodules assessed, 159 (3.5%) were classified as TI-RADS 2, 1256 (27.7%) as TI-RADS 3, 649 (14.3%) as TI-RADS 4a, 1323 (29.2%) as TI-RADS 4b and 1145 (25.3%) as TI-RADS 5. Of the 159 thyroid nodules categorized as TI-RADS 2, none were malignant (0%). Of the 1256 thyroid nodules categorized as TI-RADS 3, 52 were malignant (4.1%). Of the 649 thyroid nodules categorized as TI-RADS 4a, 78 were malignant (12.0%). Of the 1323 thyroid nodules categorized as TI-RADS 4b, 1138 were malignant (86.0%). Of the 1145 thyroid nodules categorized as TI-RADS 5, 1092 were malignant (95.4%). The calculated malignancy risk in the modified TI-RADS categories 5, 4b, 4a, 3 and 2 nodules was 95.4%, 86.0%, 12.0%, 4.1% and 0%, respectively, and all were estimated within the range of the suggested malignancy risk in the modified TI-RADS (**Table 3**).

**Table 3:** Malignancy risk according to category in the modified version of the TI-RADS.

Modified TI-RADS category	n	Malignant risk(%)	Calculated malignancy risk(%)	Frequency(%)
TI-RADS2	159	0	0(0/159)	3.5(159/4532)
TI-RADS3	1256	<5	4.1(52/1256)	27.7(1256/4532)
TI-RADS4a	649	5-15	12.0(78/649)	14.3(649/4532)
TI-RADS4b	1323	15-90	86.0(1138/1323)	29.2(1323/4532)
TI-RADS5	1145	>90	95.4(1092/1145)	25.3(1145/4532)

### Diagnostic performance of the modified version of the TI-RADS for predicting malignant thyroid nodules

**Figure 8:**
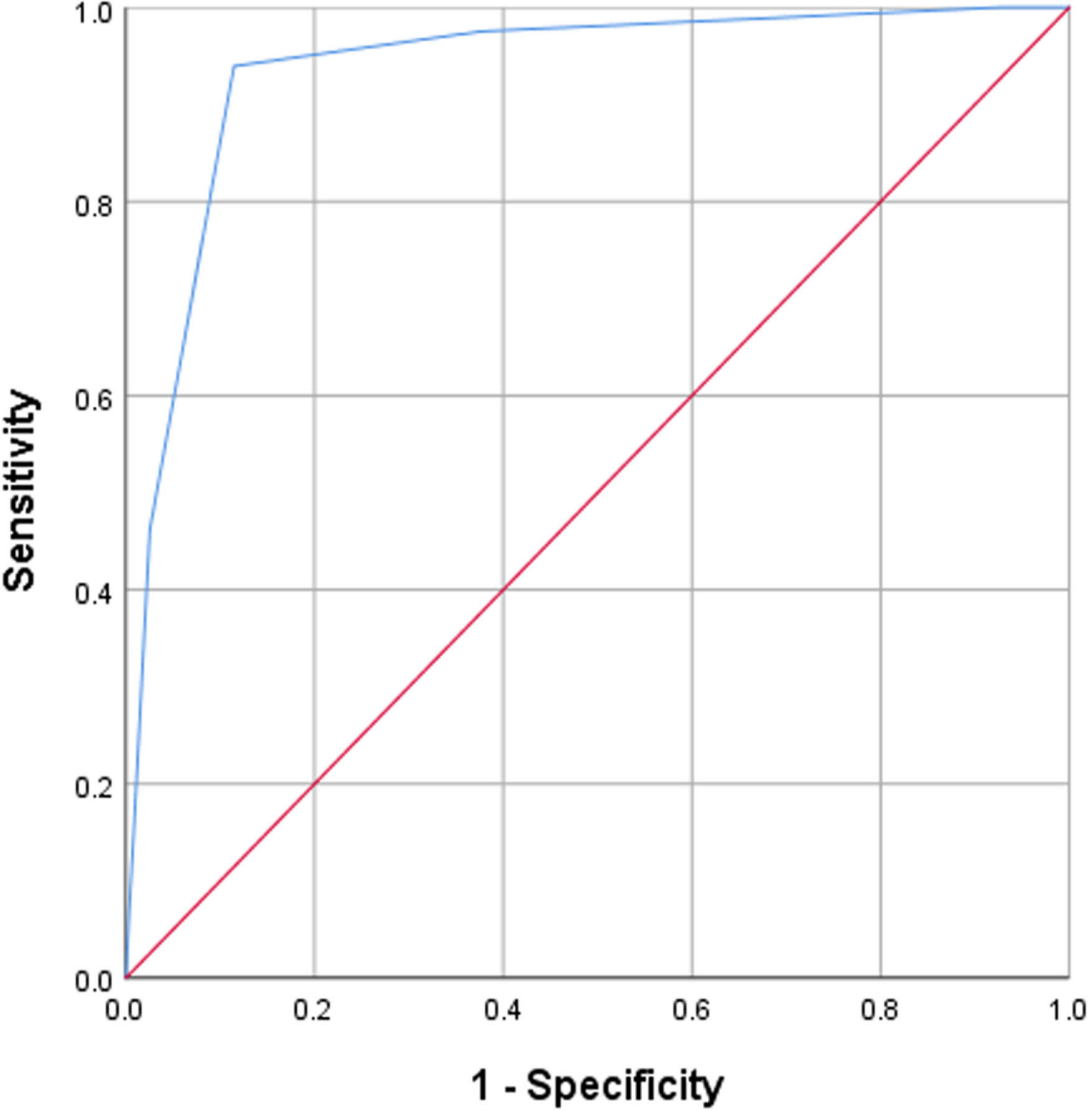
ROC analyses for the diagnostic performance of the modified version of the TI-RADS for predicting the malignancy of thyroid nodules. The best cut-off was > TIRADS 4a, resulting in 93.6% SEN and 88.5% SPN.

ROC curve analysis was used to analyze the diagnostic efficacy of the modified TI-RADS classification for differentiating benign and malignant thyroid nodules **Figure 8**. The AUC under the ROC curve was 0.936 (95% CI: 0.928–0.943, p < 0.01) and the best cut-off value for predicting malignant thyroid nodules was > TI-RADS 4a. Considering TI-RADS 4b and TI-RADS 5 together as predictors for malignancy, the SEN, ACC, PPV, NPV and SPN were 93.6%, 91.9%, 90.4%, 93.7% and 88.5%, respectively.

### Inter-observer agreement

We calculated Cohen’s Kappa value for each of the five US features and the CEUS enhancement pattern (**Table 4**). The highest inter-observer agreement was observed for the taller than wide shape and for microcalcification, the Kappa value for these two features was 0.94 and 0.93, respectively. The Kappa value for marked hypoechoic, solid composition, irregular margins and enhancement pattern of the CEUS was 0.75, 0.89, 0.86 and 0.81, respectively. There was also good agreement between the observers for the modified TI-RADS classification, the Kappa value was 0.80, thus implying that the modified TI-RADS system showed comparable results when used for the analysis of thyroid nodules by different radiologists.

**Table 4:** The inter-observer agreement for US features and TI-RADS categorization for the diagnosis of thyroid nodules.

Feature	κ coefficients	P Value
Composition	0.89	<0.001
Solid/Mixed		
Echogenicity	0.75	<0.001
Marked hypoeechoic/hyper/iso/hypoechoic		
Margins	0.86	<0.001
Well circumscribed/Irregular		
Calcification	0.93	<0.001
Microcalcifications/macrocalcifications/no calcifications		
Shape	0.94	<0.001
Taller than wide/Wider than tall		
Enhancement mode	0.81	<0.001
Ring/High/Equal/low enhancement		
Modified TI-RADS categorization	0.80	<0.001
TI-RADS 2/3/4a/4b/5		

## Discussion

TI-RADS is a quantitative scoring method that has been developed over recent years. This system can stratify the risk of malignancy for thyroid nodules and standardize the US reports for the thyroid. Consequently, this method is an effective form of communication between clinicians and pathologists. Since Horvath et al. (11) first proposed TI-RADS as a quantitative system for the risk stratification of thyroid nodules in 2009, its format and content have evolved and undergone significant development. Many researchers (12, 13, 17–19) have proposed different TI-RADS classification systems which have been used for the effective management of US for thyroid nodules. However, despite these efforts, there were many different versions and complex models of the TI-RADS classification and there was no unified classification standard. Furthermore, there were different guidelines for TI-RADS in different regions and countries, and there were certain differences between different guidelines this meant that the system was not widely adopted across the world. Therefore, there has been many attempts to develop a practical and standardized risk stratification system for thyroid nodules so as to provide consistent management strategies for assessing thyroid nodules in clinical practice (20).

When facilitated by micro-bubble contrast agents, CEUS can display microvessels, large vessels and dynamic perfusion simultaneously. Compared with conventional ultrasound, CEUS can reveal better characteristics of focal thyroid nodules (21). At present, CEUS is widely used for the differential diagnosis of benign and malignant thyroid nodules. Zhao et al. (15) showed that CEUS has high value for the differentiation of benign and malignant thyroid nodules, and was significantly more useful than conventional ultrasound. The results of the present study showed that benign thyroid nodules mainly showed ring enhancement, high enhancement or equal enhancement, while malignant nodules mainly showed low enhancement, especially non-homogeneous low enhancement, these findings were similar to those of previous studies (22). In this study, low enhancement was used as the standard to judge malignant nodules. The SEN, SPN, ACC, PPV and NPV of benign and malignant thyroid nodules diagnosed by CEUS were 82.8%, 81.6%, 82.3%, 83.0% and 81.4%, respectively. Low enhancement was considered to be the main enhancement mode for thyroid malignant nodule CEUS (23–25). Compared with the low enhancement mode of malignant nodules, benign nodules mainly showed high enhancement, equal enhancement and ring enhancement (26–28).

Many studies have shown that the combination of CEUS and TI-RADS classification for conventional ultrasound can improve the accuracy of diagnosing benign and malignant thyroid nodules. Zhao et al. (15) retrospectively analyzed the conventional ultrasound and CEUS enhancement characteristics of 117 cases of thyroid nodules and compared the diagnostic efficiency of TI-RADS alone against CEUS combined with TI-RADS for predicting benign and malignant thyroid nodules. The results showed that the ACC, SEN, SPN, PPV and NPV for TI-RADS + CEUS were the highest and were significantly higher than that for TI-RADS or CEUS alone. Ruan et al. (29) constructed a CEUS TI-RADS by adding CEUS to widely accepted nonenhanced US features, the CEUS TI-RADS showed the highest AUC under the ROC curve comparison with all other systems (AUC=0.93, P<0.001), the highest biopsy yield of malignancy at 66% (157 of 239 nodules), and the lowest unnecessary biopsy rate at 34% (82 of 239 nodules). Our previous retrospective study (17) of 298 thyroid nodules in 206 patients showed that the SEN, SPN, ACC, PPV and NPV acquired by CEUS combined with TI-RADS were 96.3%, 94.7%, 95.0%, 80.0% and 99.1%, respectively. Furthermore, the diagnostic efficiency of CEUS for judging benign and malignant thyroid nodules was significantly higher than that of TI-RADS or CEUS alone.

Many studies have stratified the risk of each TI-RADS category separately. Although there were some differences between these studies, they all reported a common pattern, with the risk of malignancy increasing from the TI-RADS 2 to the TI-RADS 5 category. The risk of malignancy described by Horvath et al. (11) was 0, < 5%, 5%–10%, 10%–80% and > 80%, respectively for TI-RADS 2, 3, 4a, 4b, and 5 lesions. Horvath et al. prospectively verified the diagnostic value of this TI-RADS classification for evaluating 1097 benign and malignant thyroid nodules. This previous study showed that the SEN, SPN, PPV, NPV and ACC were 88%, 49%, 49%, 88% and 94%, respectively. The risks of malignancy reported by Park et al. (12) was 0%–7%, 8%–23%, 24%–50%, 51%–90% and 91%–100%, respectively for TI-RADS 1, 2, 3, 4 and 5 lesions. Wang et al. (30) reported that the SEN, SPN, ACC, PPV and NPV when using Park’s TI-RADS system were 96.8%, 71.3%, 83.0%, 74.1% and 96.3%, respectively. The risks of malignancy reported by Kwak et al. (13) were 0%, 2–2.8%, 3.6–12.7%, 6.8–37.8%, 21%–91.9% and 88.7%–97.9%, respectively, for TI-RADS2, 3, 4a, 4b, 4c and 5 lesions. Zhang et al. (17) reported that the SEN, SPN, PPV, NPV and ACC were 94.4%, 69.3%, 40.5%, 98.3% and 73.8%, respectively. In our present study, the calculated risk of malignancy for the modified TI-RADS categories 5, 4b, 4a, 3 and 2 nodules were 95.4%, 86.0%, 12.0%, 4.1% and 0%, respectively. The risk of malignancy risk for the five categories was within the range of the suggested risk of malignancy. Therefore, the modified TI-RADS can be applied for the qualitative diagnosis of benign and malignant thyroid nodules.

Based on ROC curve analyses, our study revealed an AUC of 0.936, the best cut-off value for predicting malignant thyroid nodules was > TI-RADS4a. When considering TI-RADS 4b and TI-RADS 5 together as predictors for malignancy, the SEN, ACC, PPV, NPV and SPN were 93.6%, 91.9%, 90.4%, 93.7% and 88.5%, respectively. In our study, the modified TI-RADS had high diagnostic efficiency for the diagnosis of benign and malignant thyroid nodules. Of the 4352 thyroid nodules assessed, 159 were classified as TI-RADS 2, none were malignant and the diagnostic accordance rate was 100%. In total, 1256 were classified as TI-RADS 3, of which 1204 were benign nodules; the diagnostic accordance rate was 95.9%. In total, 649 were classified as TI-RADS 4a, of which 571 were benign nodules; the diagnostic accordance rate was 88.0%. In total, 1323 were classified as TI-RADS 4b, of which 1138 were malignant nodules; the diagnostic accordance rate was 86.0%. In total, 1145 were classified as TI-RADS 5, of which 1092 were malignant nodules; the diagnostic accordance rate was 95.4%. Except for TI-RADS 2 nodules, there was a certain misdiagnosis rate in other classified nodules. There are several potential reasons for these findings. First, as with conventional two-dimensional ultrasound, CEUS enhancement patterns for some benign and malignant nodules can also overlap. For example, some malignant nodules had a rich blood supply and showed high enhancement, while some benign nodules may exhibit scar hyperplasia or fibrous tissue hyperplasia, thus resulting in low enhancement. Second, some nodules were so small that it was difficult to judge their CEUS enhancement mode. Third, some benign nodules were often associated with focal PTC or FTC carcinogenesis; therefore, it was difficult to correctly diagnose these focal forms of carcinogenesis. Fourth, the presence of hidden PTMC in thyroid glands with diffuse lesions was not typical on conventional two-dimensional ultrasound or CEUS, thus increasing the difficulty of diagnosis. Finally, some thyroid inflammatory lesions were similar to malignant lesions in conventional two-dimensional ultrasound or CEUS enhancement mode; it was difficult to correctly diagnose these inflammatory lesions.

The modified TI-RADS is simple and accurate for the evaluation of benign and malignant thyroid nodules. The radiologists only need to accurately evaluate the conventional two dimensional ultrasound signs and CEUS enhancement mode to classify nodules. Theoretical malignant risk and clinical treatment suggestions were also given for each classification of nodules, thus allowing better communication between clinicians and pathologists. Another important role of the modified TI-RADS was to standardize the criteria for different radiologists to evaluate the signs of thyroid nodules. Therefore, the classification system should have good consistency and repeatability among different radiologists (31–33). Our study revealed strong inter-observer agreement between different radiologists when using the modified TI-RADS categories and features for thyroid nodule characterization. We found that the highest inter-observer agreement was for shape and micro-calcification. The Kappa value for these two features was 0.94 and 0.93, respectively, thus showing excellent consistency among the different observers. There was also good consistency among the observers for echogenicity, composition, margins and enhancement mode; the Kappa values were 0.75, 0.89, 0.86 and 0.81, respectively. In addition, different observers showed good consistency when using the modified TI-RADS classification system; the Kappa value was 0.80. These results showed that the modified TI-RADS produced comparable results for the analysis of suspicious thyroid nodules when used by different radiologists in thyroid imaging.

This study had some limitations that need to be considered. First, all US examinations were performed and analyzed by highly experienced radiologists. Further studies relating to the performance of this reporting system when applied by less experienced radiologists may be needed. Second, we did not compare the modified TI-RADS to the other TI-RADS classification systems. Third, we only evaluated inter-observer consistency, we did not evaluate intra-observer consistency, this needs to be addressed by future studies.

## Conclusion

The modified TI-RADS had high diagnostic efficiency for the diagnosis of benign and malignant thyroid nodules. The inter-observer agreement for the modified TI-RADS category was excellent, thus suggesting that this classification is very suitable for clinical application.

## Data availability statement

The raw data supporting the conclusions of this article will be made available by the authors, without undue reservation.

## Ethics statement

Written informed consent was obtained from the individual(s) for the publication of any potentially identifiable images or data included in this article.

## Author contributions

PiZ and FC have equal contributions to this article. Drafting the work or revising: WL, PiZ. Acquisition, analysis, or interpretation of data: WL, FC, PeZ, LX, LW, ZW, YY, XL, BW, WY, HZ, YT. Conception or design: WL, PiZ. Final approval of the manuscript: PiZ.
